# Efficacy and safety of combination cream of stimu-tex AS™ (spent grain wax, Argania Spinosa Kernel oil, Butyrospermum Parkii (shea butter) extract), and saccharide isomerate after fractional CO2 laser procedure: split-face, double blinded, randomized controlled trial

**DOI:** 10.1007/s10103-025-04703-5

**Published:** 2025-10-24

**Authors:** Irma Bernadette S. Sitohang, Abraham Arimuko, Lilik Norawati, Rita Maria, Cyntia Yulyana, Randy Satria Nugraha, Vashty Amanda, Arsy Indrafatina, Inasa Amalia

**Affiliations:** 1 Division of Cosmetic Dermatology, Department of Dermatology and Venereology, Faculty of Medicine, Universitas Indonesia, Dr. Cipto Mangunkusumo Hospital, Jakarta, Indonesia; 2 Medical Staff Unit of Dermatology and Venereology, Faculty of Medicine, Universitas Indonesia, Universitas Indonesia Hospital, Depok, Indonesia; 3 Department of Dermatology and Venereology, Presidential-Army Central Hospital Gatot Soebroto, Jakarta, Indonesia; 4 Indonesia Cosmetic Dermatology Study Group, Department of Dermatology and Venereology, Presidential-Army Central Hospital Gatot Soebroto, Jakarta, Indonesia

**Keywords:** Fractional CO_2_ laser, Wound healing, Erythema, Skin capacitance, TEWL

## Abstract

Until now, fractional CO2 laser has been the gold standard for photodamage skin treatment. Topical therapy modalities can accelerate the wound healing process and influence the treatment results. However, no standardized topical therapy exists for subjects after fractional CO2 laser procedures. This research aims to determine the efficacy and safety of a cream combination of Stimu-tex AS™ (Spent Grain Wax, Argania Spinosa Kernel Oil, Butyrospermum Parkii (Shea Butter Extract) and Saccharide Isomerate after fractional CO2 laser treatment. A split-face, double-blinded, and randomized study was conducted in 2021 with 20 subjects, each given two types of creams, A and B, following fractional CO2 laser procedure. Subjects will apply cream A and cream B to each side of the face according to the randomization results. One of the creams was a moisturizing combination cream with a combination of Stimu-tex AS™ (Spent Grain Wax, Argania Spinosa Kernel Oil, Butyrospermum Parkii (Shea Butter Extract), and Saccharide Isomerate. Meanwhile, another cream was a placebo. The outcome assessment of this research was carried out subjectively and objectively. The subjective assessment was evaluated using the visual analogue scale (VAS). In contrast, the objective assessment evaluated the degree of erythema using the clinical erythema assessment score (CEA), dermoscopic examination, transepidermal water loss (TEWL) examination with TEWAmeter, and skin capacitance (SCap) with corneometer. This assessment was carried out before the laser procedure, 15 min, on the third day, and ^h^ the seventh day after the laser procedure. There are 20 subjects recruited into the study, with a split face method, resulting in 40 sample sizes. Significant differences between the experimental and placebo groups were obtained in the assessing of the CEA scale on the third day after the procedure. Meanwhile, on the seventh day after the procedure, significant results were obtained on the TEWL examination with TEWAmeter, SCap with Corneometer, and the degree of erythema using the CEA score. Apart from that, each group had no complaints about adverse events or serious adverse events (SAEs). The combination cream provides good results after the fractional CO2 laser procedure, by reducing the degree of erythema, increasing SCap, and reducing TEWL. Furthermore, this combination cream is safe to use because there were no reports of SAEs.

## Key messages

- A combination cream containing Stimu-tex AS^TM^ (Spent Grain Wax, Argania Spinosa Kernel Oil and Butyrospermum Parkii (Shea Butter) Extract) and Saccharide Isomerate provides a moist environment to accelerate wound healing and reduce erythema after fractional CO2 laser procedure.
- This split-face, double-blind randomized clinical trial aims to determine the efficacy and safety of a cream combination of Stimu-tex AS^TM^ (Spent Grain Wax, Argania Spinosa Kernel Oil, Butyrospermum Parkii (Shea Butter Extract) and Saccharide Isomerate versus placebo after fractional CO2 laser treatment within 20 subjects
- This combination cream has been proven to increase skin hydration, reduce TEWL, and reduce erythema. There are no complaints, adverse events, and serious Adverse Events that arise when using this combination cream, thus this combination cream is safe to use.

## Introduction

A fractional CO2 laser with a wavelength of 10:600 nm is a modality used for wrinkles, large pores, scars, stretch marks, and benign skin tumors. Water is the chromophore target in this laser. However, a fractional CO2 laser has several side effects that can be serious, although rare, including thermal injury, risk of depigmentation and scarring, persistent erythema, and the need to avoid long-term sun exposure [1]. 

The aim of using moisturizers is to hydrate the skin, maintain suitable skin barriers [2], relieve symptoms, cause fast and efficient healing in the superficial structures of the epidermis, namely the stratum corneum [3], and minimize the risk of infection [4]. However, no standardized moisturizer has been used after the fractional CO2 laser procedure. The combination cream used is a moisturizer enriched with Stimu-tex AS™ (Spent Grain Wax, Argania Spinosa Kernel oil, and Butyrospermum Parkii (Shea Butter) Extract) and Saccharide Isomerate.

Therefore, the airm of this study is to determine the efficacy and safety of moisturizing cream containing a combination of Stimu-tex AS™ and Saccharide Isomerate after fractional CO2 laser treatment.

## Materials and methods

This research has received ethical approval from the Ethics Commission of the Faculty of Medicine, Universitas Indonesia, with ethical number KET-1130/UN2.F1/ETIK/PPM.00.02/2021. All participants were informed about the study procedures and provided written informed consent prior to participation. The study was conducted in accordance with the principles of the Declaration of Helsinki. This trial was registered at ClinicalTrials.gov (registration number: NCT05186246).

### Subjects

This study was conducted on 20 subjects at the Department of Dermatology and Venereology, Presidential-Army Central Hospital Gatot Soebroto, Jakarta, Indonesia, in 2021. The eligibility criteria included women aged 18–60 years who visited the Dermatology and Venereology Clinic for a fractional CO2 laser procedure. The subjects have or will receive a minimum priming containing topical retinoic acid at a concentration of 0.05% tretinoin cream for at least two weeks before the laser procedure, and the subjects understand and agree to participate.

Exclusion criteria included: (1) Pregnant women, breastfeeding, or taking oral contraceptives at the time of examination. (2) History of consuming systemic retinoids in the previous 3 months. (3) History of suffering from or undergoing therapy for hormonal or endocrine disorders or other serious illnesses. (4) Currently on immunosuppressant therapy. (5) Non-compliance with treatment.

Additionally, participants with a history of impaired wound healing, such as diabetes mellitus or other healing disorders, should also be excluded, as these conditions can significantly affect study outcomes.

### Procedure

**Fig. 1:**
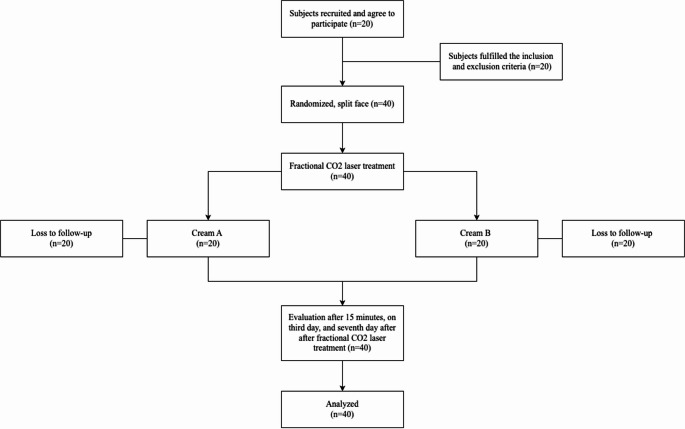
Clinical trial flowchart

Each subject was given information about the research and filled out an informed consent form. Each subject underwent anamnesis, physical examination, clinical documentation, visual analogue scale (VAS) assessment, the degree of erythema using the clinical erythema assessment (CEA) scale, dermoscopic examination, and transepidermal water loss (TEWL) examination with a TEWAmeter and skin capacitance (SCap) with a Corneometer at each visit. At the initial visit, subjects were given Retinoic Acid cream 0.05% and instructed to use it for 14 days. On the second visit, the subject underwent fractional CO₂ laser treatment on the entire face using the S-CO₂ fractional laser device (DANA, South Korea) with the following parameters: energy 37.5 mJ, pulse duration 1.5 ms, and distance 1 mm. After the procedure, the subject’s face was compressed with normal saline (0.9% NaCl) for 20 min and dried. After that, VAS assessment, the degree of erythema using CEA score, Dermoscopic examination, TEWL examination with TEWAmeter, and SCap examination with Corneometer were assessed. Cream A and B were applied to each side of the subject’s face using a split-face study according to the randomization results, as shown in Fig. 1. One of the creams was a moisturizing combination cream with a combination of Stimu-tex AS™ and Saccharide Isomerate.

The placebo cream was formulated using standard excipients with no active pharmaceutical ingredient. the formulation is comprised of *Paraffinum liquidum*, *Cetaceum*, *Acidum stearicum*, *Cera alba*, Triethanolamine, Glycerin, Parfum, and *Aqua ad*. The Placebo vehicle was selected as the control to avoid the confounding effects of active agents and to allow a direct comparison with intervantion moisturizing cream.

Meanwhile, the other cream was a placebo. Subjects were provided daily records about the application times of both creams on each side of the face and any occurring side effects. The creams were used twice daily, in the morning and evening. Creams were applied for seven days. This assessment was carried out before the laser procedure, 15 min, on the third day, and seventh day after laser procedure.

### Examinations

All examinations were conducted in a well-lit room after using a facial cleaner. The severity and affected area of the erythema were assessed and graded on the 1–4 Clinician Erythema Assessment (CEA) scale and documented using a Canon EOS 3000D DSLR camera, based on visual inspection by the two dermatologists who served as investigators. To minimize inter-observer variability, discrepancies were resolved by consensus. Transepidermal water loss (TEWL) was measured with the TEWAmeter^®^ TM 210 probe (Courage + Khazaka Electronic GmbH, Cologne, Germany), which was linked to the MPA system interface. Measurements for each location, specifically the cheek and forehead, were conducted three times, and the average values were recorded. Dermoscopy (Heine Delta 30) examination was conducted to observe inflammation, focusing on vascular patterns, pigmentary changes, erythema decrease, and progressive re-epithelialization.

### Evaluation

Evaluation was carried out by assessing subjectivity with VAS and assessing objectivity by evaluating the degree of erythema using CEA score, dermoscopic examination, TEWL examination with TEWAmeter, and SCap with a corneometer on both sides of each subject’s face. The examination is carried out by facial inspection. Evaluation was carried out 15 min (baseline), 3rd day and 7th day after the fractional CO2 laser procedure.

### Statistical analysis

Randomization blinding code was kept by the statistical consultant. The masking code is revealed at the completion of the study for statistical analysis. The data obtained was analyzed using the SPSS version 21.0 application. Data were analyzed using the paired T-test and or the Wilcoxon signed-rank test as a non-parametric alternative.

## Results

The most significant number of subjects were Javanese with 13 people, followed by Sundanese with three subjects, and Batak, Betawi, Minangkabau, and Sundanese-Javanese with one person each. The mean age of the subjects was 42 years (with a range of 29–58 years). Skin aging was found in 14 subjects, with 10 of them also experiencing other skin conditions. Among them, five subjects had acne scars, and four of them also had other skin abnormalities such as depigmentation, melasma, or uneven skin tone. The initial characteristics of the research subjects can be seen in Table 1.

**Table 1:** Initial clinical characteristics of subjects

Variable	Experimental Group	Placebo Group	*P*-value
TEWAmeter on Cheek, mean (SD)	71,63 (16,7)	73,63 (14,7)	0,6889
TEWAmeter on Forehead, mean (SD)	69,07 (15,2)	69,43 (14,1)	0,9386
Corneometer on Cheek, mean (SD)	37,46 (9,9)	37,33 (8,8)	0,9662
Corneometer on Forehead, mean (SD)	38,46 (14,1)	43,43 (13,2)	0,2567
Pores	6,59 (1,0)	6,59 (1,0)	1,000
Wrinkles	3,94 (1,2)	3,94 (1,2)	1,000
Pigmentation	6,21 (0,9)	6,21 (0,9)	1,000
Sebum	671,11 (422,7)	671,11 (422,7)	1,000

*SD* standard deviation

**Fig. 2:**
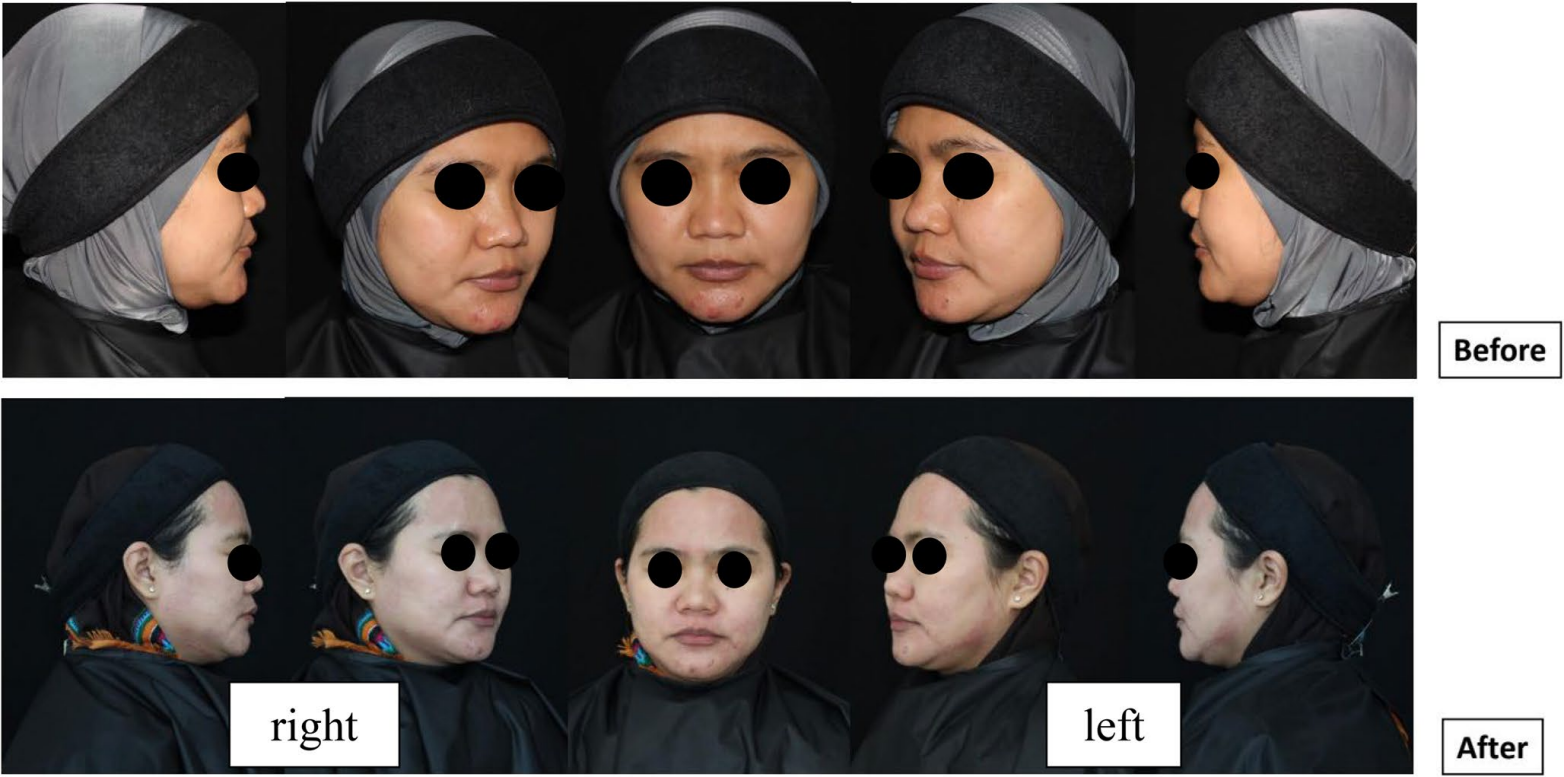
Comparison of split-face outcomes in patients before and at day 3 after fractional CO₂ laser treatment: right side treated with the experimental cream, and left side treated with the placebo cream showed more erythema

TEWAmeter and Corneometer examinations on the 3rd day after the procedure did not show any significant differences in results between the experimental group and the placebo group (Table 2)**.** On the 7th day after the procedure, there were significant differences between the experimental and placebo groups in the TEWAmeter examination on the cheeks (*p* = 0.0187) and on the forehead (*p* = 0.0014). Significant differences on the 7th day were also found in the results of Corneometer examinations on the cheeks (0.0008) and on the forehead (0.0041). There were no significant differences in complaints or adverse events in the two groups. The results of the erythema assessment on the 3rd day and 7th day after the procedure found significant differences, respectively (*p* = 0.002) and (*p* = 0.004). On the 7th day, there were more subjects without erythema (grade 0) in the experimental group compared to the placebo group, namely 75%, while there were more subjects with grade 1 erythema in the placebo group than the experimental group, namely 70%. These clinical differences can also be observed in Fig. 2. There were no significant differences in the results of examining pores, wrinkles, pigment and sebum between the experimental group and the placebo group at each visit.

**Table 2:** The examination results on the third and seventh day after the procedure

Variable		Third day			Seventh day		
		Experimental	Placebo	*P*- value	Experimental	Placebo	*P*- value
TEWAmeter Cheek, mean (SD)		21,14 (5,4)	23,88 (6,9)	0,0852	15,64 (4,9)	20,08 (1,7)	**0**,**0187**
TEWAmeter Forehead, mean (SD)		23,15 (13,8)	30,81 (21,4)	0,0931	18,07 (7,2)	32,83 (19,4)	**0**,**0014**
Corneometer Cheek, mean (SD)		26,34 (13,9)	24,83 (12,6)	0,3599	43,47 (9,5)	32,83 (10,4)	**0**,**0008**
Corneometer Forehead, mean (SD)		33,38 (16,1)	32,87 (13,5)	0,4566	48,36 (13,8)	36,05 (14,2)	**0**,**0041**
Complaint, n (%)	0	17 (85,00)	15 (75,00)	0,695	19 (95,00)	18 (90,00)	1,000
	1	3 (15,00)	5 (25,00)		1 (5,00)	2 (10,00)	
Adverse Events, n (%)	0	13 (65,00)	11 (55,00)	0,749	18 (90,00)	17 (85,00)	1,000
	1	5 (25,00)	5 (25,00)		1 (5,00)	2 (10,00)	
	2	2 (10,00)	4 (20,00)		1 (5,00)	1 (5,00)	
CEA, n (%)	0	1 (5,00)	1 (5,00)	**0**,**002**	15 (75,00)	5 (25,00)	**0**,**004**
	1	14 (70,00)	4 (20,00)		5 (25,00)	14 (70,00)	
	2	5 (25,00)	15 (75,00)		0 (0,00)	1 (5,00)	

*CEA* Clinical Erythema Assessment (0 : Clear, 1 : Almost Clear, 2 : Mild, 3 : Moderate, 4 : Severe), *n* number, *SD* standard deviation

The study found that side effects were minor and similar in both groups. Mild reactions occurred in 25% of participants, affected 10% of the treatment group and 20% of the placebo group. There were no significant in side effects.

## Discussion

Photodamaged skin can be treated with various procedures, including chemical peels [5]. Just like chemical peeling and microneedling, fractional CO2 lasers can be utilized to address photodamage skin, hypertrophic, hypotrophic, or atrophic scars [6, 7]. However, the fractional CO2 laser procedure is the gold standard for treating photodamaged skin. Fractional CO2 laser procedure is carried out in several stages. The subjects applied a minimum priming containing topical retinoic acid of 0.05% for at least two weeks before the laser procedure. Topical retinoic acid (0.05% all-trans-retinoic acid) improves skin rejuvenation through increased elasticity, wrinkle amelioration, and pigmentation enhancement. It stimulates dermal thickness, hydroxyproline content, and type III collagen expression, with combination therapy using fractional CO₂ laser producing the greatest effects. This combination leverages the rapid impact of laser treatment and the sustained efficacy of retinoic acid, further improving and prolonging skin rejuvenation results [37]. After the procedure, subjects were instructed to use high-spectrum sunscreen and moisturizer during the day [3]. 

The results of this therapy are influenced by wound healing process through post-laser therapy. The recommended treatment is moisturizer. Unfortunately, there is no standardized moisturizer which is used as a standard post-laser therapy. A case series was reported by Lestari et al. They used a combination cream of panthenol, madecassoside, and niacinamide after Q-switched Nd: YAG laser therapy. Erythema reduced after day 7, but further research is needed [8]. 

The combination cream with Stimu-tex AS™ contains unsaturated fatty acids such as linoleic acid (omega-6), oleic acid, stearic acid, and palmitic acid. Linoleic acid has an antiinflammatory effect [9, 10] and can repair damaged skin barriers [11]. In addition, three extracts can relieve epidermal irritation and allergy symptoms and reduce the release of histamine, reducing irritation and itching [12]. Leeyaphan et al. reported that the use of a combination Stimu-tex AS™ is safe and can be used [13]. 

Butyrospremum Parkii (Shea Butter) Extract has the main content, namely triglycerides, consisting of oleic acid, linoleic acid, stearic acid, and palmitic acid [14]. Other contents are tocopherols, sterols, and phenols. The high tocopherol content in shea butter can increase the regulation of ceramide production [15] and function as an antioxidant [14]. In Shea Butter extract, triterpenes are also found to be moisturizing and anti-aging [16], anti-inflammatory [17], antioxidant[18–22], and antitumor effects [18, 19]. The cinnamate esters contained in it also have anti-inflammatory effects [18]. Besides that, shea butter also has antimicrobial [19–25], antifungal, anti-viral [26], anti-cancer effects[27], and at the cellular level, it can inhibit several inflammatory pathways by inhibiting COX-2 and iNOS, downregulating the production of TNF-α, IL-1ꞵ, and IL-12 [28]. Elsewedy et al. reported that Shea Butter was safe to use without causing irritation and side effects [29]. 

Argania Spinosa Kernel (Argan) oil contains 80% unsaturated fatty acids. It contains tocopherols, sterols, polyphenols, squalene, and triterpenes. Topical application can increase skin elasticity [30]. Apart from playing a role in skin elasticity, argan oil can accelerate wound healing and act as an anti-inflammatory [30, 31]. Argan oil can also increase skin hydration by improving the skin barrier and maintaining water-holding capacity [32, 33]. No serious adverse events (SAEs) occurred during the study by Boucetta et al., so the argan oil is safe to use [32]. 

Saccharide Isomerate has several benefits, such as maintaining moisture and increasing the water content in the stratum corneum [33, 35]. Dewi et al. reported that the adding of 5% Saccharide isomerate to the moisturizer formula can improve and maintain skin hydration [36]. Hartini et al. found that using moisturizer containing Saccharide Isomerate significantly reduces TEWL [35]. Apart from maintaining skin hydration, Saccharide Isomerate can also be used as anti-aging [36]. 

The results of the corneometer on the third day following the laser procedure showed that corneometer results on the forehead in the experimental group were better than those of the placebo group. However, there was no significant difference between them. In contrast, on the seventh day post-laser treatment, the results for both forehead and cheek corneometer in the experimental group were better than those in the placebo group. Significant results were obtained on the forehead and cheeks with *p-values* ​​of 0.0041 and 0.0008 respectively. The results showed that the combination cream hydrates the skin more than the placebo cream, with various ingredients that can hydrate the skin, such as Shea Butter [16], Argan Oil [32, 33], and Saccharide Isomerates [34–36]. 

The result on the third day of the TEWAmeter examination, showed improvements in TEWL in both the experimental and the placebo groups, compared with the TEWAmeter results before the laser procedure. The experimental group had better results on the third day after the laser procedure. Unfortunately, there was no significant difference compared with the placebo group. On the seventh day, it was found that the results in the experimental group were excellent because it showed that the skin condition was expected, with an average TEWL of the forehead and cheeks of 18.07 and 15.64, respectively. Meanwhile, in the placebo group, the average forehead TEWL result was worse (32.83), considered a critical skin condition. Thus, the combination cream is more beneficial than the placebo cream. Apart from that, there were also significant results when using the combination cream. These results align with the previous finding of Hartini et al., which was that Saccharide Isomerates significantly reduced TEWL [35]. 

Evaluation of erythema using CEA score on the third and seventh day showed significant results between the experimental and placebo groups, with *p-values* ​​of 0.002 and 0.004. On the third day, erythema occurred more frequently in the placebo group. Likewise, on the 7th day, excellent results were obtained in the experimental group, with erythema grade 0 found at 75% of subjects and grade 1 at 25%. Meanwhile, erythema grade 0 was 25% of subjects in the placebo group, and erythema grade 1 was 70%. These outcomes supported the idea that the combination cream has an anti- inflammatory effect obtained from the Grain Spent Wax, Argan Oil, and Shea Butter [10, 11, 17, 28]. The antioxidant effect is also obtained from the shea butter content [14, 18, 19] The excellent improvement in erythema on the seventh day is also one of the benefits of Argan Oil, which accelerates wound healing [30, 31]. 

No significant results were found between the experimental and the placebo groups regarding complaints or adverse events on the third or seventh day. The absence of complaints, significant adverse events, and SAEs in this combination cream is by Jirabundansuk et al. that Spent Grain Wax, Argan Oil, and Shea Butter can reduce histamine release [10]. With the absence of complaints, adverse events, and SAEs, it can be concluded that using a combination cream containing Stimu-tex AS™ and Saccharide Isomerate is safe.

This study had some strengths. First, randomization and double-blind techniques were used to ensure objective and unbiased results. In addition, at the beginning of the study, an evaluation was carried out regarding the initial characteristics of the subject’s skin using anamnesis, physical examination, dermoscopic examination, TEWAmeter, and corneometer. However, further studies are recommended to complete this study such as larger sample size and including various population subjects with Fitzpatrick types I-VI.

## Conclusion

The use of a combination cream containing Stimu-tex AS™ and Saccharide Isomerate provides a moist environment to accelerate wound healing and reduce erythema after fractional CO2 laser procedure. This combination cream has been proven to increase skin hydration, reduce TEWL, and reduce erythema. There are no complaints and Serious Adverse Events (SAEs) that arise when using this combination cream, thus this combination cream is safe to use.

### Limitations

This research has several limitations. First, the sample size was quite small (*n* = 20), which may limit the statistical power and generalizability of the findings. Second, most participants in the study were Asian, thereby limiting the findings’ applicability to other ethnic demographics. Lastly, the follow-up duration was restricted to seven days, which prevented an assessment of long-term outcomes, such as recurrence or sustained effectiveness.

## Data Availability

Data supporting the findings of this study are available from the corresponding author upon reasonable request. The clinical trial was registered at ClinicalTrials.gov with the registration number NCT05186246.

## Declarations

### **Clinical trial number**

This trial was registered at ClinicalTrials.gov (registration number: NCT05186246). 

## References

[1] Petrov A (2016) Efficiency of carbon dioxide fractional laser in skin resurfacing. Open Access Maced J Med Sci 4(2):271–276 27335599 10.3889/oamjms.2016.062 PMC4908744

[2] Neogenesis Fractional lasers – fraxel, CO2. [Cited 2022 Feb 21]. Available from: https://www.neogenesispro.co.uk/fractional-lasers-fraxel-co2/

[3] Santos-Caetano JP, PharmB RV, Gfeller CF, Cargill M, Mhalingam H (2019) Cosmetic use of three topical moisturizers following glycolic acid facial peels. J Cosmet Dermatol 00:1–11 10.1111/jocd.13074 31322804

[4] Ramsdell WM (2012) Fractional CO2 laser resurfacing complications. Semin Plast Surg 26:137–140 23904822 10.1055/s-0032-1329415 PMC3580977

[5] Sitohang IB, Rahmayunita G, Hosfiar VA, Ninditya S, Augustin M (2021) Effectiveness of water as the neutralising agent for glycolic acid peels in skin phototypes IV-V. Australas J Dermatol 62(2):e212–e216. 10.1111/ajd.13486 33070326 10.1111/ajd.13486

[6] Sitohang IB, Sirait SAP, Suryanegara J (2021) Microneedling in the treatment of atrophic scars: a systematic review of randomised controlled trials. Int Wound J 18(5):577–585. 10.1111/iwj.13559 33538106 10.1111/iwj.13559 PMC8450803

[7] Sitohang IB, Sirait SA, Safira FD (2022) Fractional carbon dioxide laser for treating hypertrophic scars: A systematic review of randomised trials. Australas J Dermatol 63(1):27–35. 10.1111/ajd.13730 34628639 10.1111/ajd.13730

[8] Lestari AA, Adistri K, Hopkop T, Sitohang IB (2023) Combination of panthenol, madecassoside and niacinamide in multi-lamellar emulsion as postprocedural laser treatment. J Pak Assoc Dermatol 33(2):706–710

[9] Calder PC (2005) Polyunsaturated fatty acids and inflammation. Biochem Soc Trans 33:423–7 15787620 10.1042/BST0330423

[10] Jirabundansuk P, Ophaswongse S, Udompataikul M (2014) Comparative trial of moisturizer containing spent grain wax, *Butyrospermum parkii* extract, *Argania spinosa* kernel oil vs. 1% hydrocortisone cream in the treatment of childhood atopic dermatitis. J Med Assoc Thai 97(8):820–826 25345257

[11] Elias PM, Brown BE, Ziboh VA (1980) The permeability barrier in essential fatty acid deficiency: evidence for a direct role for linoleic acid in barrier function. J Invest Dermatol 74(4):230–233. 10.1111/1523-1747.ep12541775 7373078 10.1111/1523-1747.ep12541775

[12] Hon KL, Wang SS, Pong NH, Leung TF (2013) The ideal moisturizer: a survey of parental expectations and practice in childhood-onset eczema. J Dermatolog Treat 24:7–12. 10.3109/09546634.2012.672713 22390400 10.3109/09546634.2012.672713

[13] Leeyaphan C, Varothai S, Trakanwittayarak S et al (2022) A randomized controlled trial to compare the effectiveness and safety of adsorbent lotion containing tapioca starch, spent grain wax, *Butyrospermum parkii* extract, *Argania spinosa* kernel oil, *Aloe barbadensis*, rosehip oil, and allantoin with a low-potency topical corticosteroid in the treatment of intertrigo. J Cosmet Dermatol 21(2):679–688. 10.1111/jocd.14125 33811776 10.1111/jocd.14125

[14] Maranz S, Wiesman Z (2004) Influence of climate on the tocopherol content of Shea butter. J Agric Food Chem 52:2934–2937 15137838 10.1021/jf035194r

[15] Lin TK, Zhong L, Santiago JL (2017) Anti-inflammatory and skin barrier repair effects of topical application of some plant oils. Int J Mol Sci 19(1):70 29280987 10.3390/ijms19010070 PMC5796020

[16] Alander J (2004) Shea butter-a multifunctional ingredient for food and cosmetics. Lipid Technol 16:202–205

[17] Lim D, Bae S, Oh T (2021) Anti-inflammatory effect of shea butter extracts in canine keratinocytes. J Veterinary Clin 83:27–31

[18] Akihisa T, Kojima N, Kikuchi T, Yasukawa K, Tokuda H, Masters T et al (2010) Anti-inflammatory and chemopreventive effects of triterpene cinnamates and acetates from Shea fat. J Oleo Sci 59:273–280 20484832 10.5650/jos.59.273

[19] Kao JH, Lin SH, Lai CF, Lin YC, Kong ZL, Wong CS (2016) Shea nut oil triterpene concentrate attenuates knee osteoarthritis development in rats: evidence from knee joint histology. PLoS ONE 11(9):e0162022 27583436 10.1371/journal.pone.0162022 PMC5008785

[20] Foyet HS, Tsala DE, Bodo JZ, Carine AN, Heroyne LT, Oben EK (2015) Anti-inflammatory and anti-arthritic activity of a methanol extract from *vitellaria paradoxa* stem bark. Pharmacogn Res 7:367 10.4103/0974-8490.159569 PMC4660517 26692752

[21] Olasunkanmi OO, Akinpelu DA, Adeniyi PO, Ajayi OF, Omololu-Aso J, Olorunmola FO (2018) Investigations into antibacterial, phytochemical and antioxidant properties of *vitellaria paradoxa* (Gaertn.) stem bark extracts. Journal of Pharmaceutical Research International 20(5):1–17

[22] Igbeneghu OA (2013) The antimicrobial assessment of some Nigerian herbal soaps. Afr J Tradit Complement Altern Med 10(6):513–518 24311879 10.4314/ajtcam.v10i6.21 PMC3847394

[23] Ojo O, Kengne MH, Fotsing MC, Mutlane EM, Ndinteh DT (2021) Traditional uses, phytochemistry, pharmacology and other potential applications of *Vitellaria paradoxa* Gaertn. (Sapotaceae): a review. Arab J Chem 14:103213

[24] Ajijolakewu KA, Awarun FJ (2015) Comparative antibacterial efficacy of *Vitellaria paradoxa* (Shea butter Tree) extracts against some clinical bacterial isolates. Not Sci Biol 7(3):264–268

[25] Wada NM, Muhammad MI, Garba USA, Ghazali HM (2019) Antibacterial activity of *vitellaria paradoxa* seed oil extract and honey against bacterial isolates from wound infection. Int J Biol Phys Chem Stud 1:16–21

[26] Boyejo I, Azeez S, Owolabi A, Issah O (2019) Antifungal and phytochemical screening of extract from *Vitellaria paradoxa* (shea butter tree) leaves, barks and roots on dermatophytes. Int J Sci Res Publications 9:90129

[27] Zhang J, Li D, Lv Q, Ye F, Jing X, Masters ET et al (2018) Compositions and melanogenesis inhibitory activities of the extracts of defatted Shea (Vitellaria paradoxa) kernels from seven African countries. J Food Compos Anal 70:89–97

[28] Verma N, Chakrabarti R, Das RH, Gautam HK (2012) Anti-inflammatory effects of Shea butter through Inhibition of iNOS, COX-2, and cytokines via the Nf-κB pathway in LPS-activated J774 macrophage cells. J Complement Integr Med 9:1–11 22499721 10.1515/1553-3840.1574

[29] Elsewedy HS, Shehata TM, Soliman WE (2022) Shea butter potentiates the anti-bacterial activity of fusidic acid incorporated into solid lipid nanoparticles. Polymers (Basel) 14(12):2436. 10.3390/polym14122436 35746012 10.3390/polym14122436 PMC9228747

[30] Boucetta KQ, Charrouf Z, Aguenaou H, Derouiche A, Bensouda Y (2015) The effect of dietary and/or cosmetic Argan oil on postmenopausal skin elasticity. Clin Interv Aging 10:339–349 25673976 10.2147/CIA.S71684 PMC4321565

[31] Avsar U, Halici Z, Akpinar E, Yayla M, Avsar U, Aaron U et al (2016) The effects of Argan oil in second-degree burn wound healing in rats. Ostomy Wound Manage 62:26–34 26978857

[32] Tichota DM, Silva AC, Sousa Lobo JM, Amaral MH (2014) Design, characterization, and clinical evaluation of Argan oil nanostructured lipid carriers to improve skin hydration. Int J Nanomed 9:3855–3864. 10.2147/IJN.S64008 10.2147/IJN.S64008 PMC4137996 25143733

[33] Vlorensia, Hartini H, Abdullah H, Martinus AR, Ikhtiari R (2019) The effect of a moisturizing cream with saccharide isomerates and ceramide on increasing skin hydration. SCITEPRESS 428–435

[34] Boucetta KQ, Charrouf Z, Derouiche A, Rahali Y, Bensouda Y (2014) Skin hydration in postmenopausal women: Argan oil benefits with oral and/or topical use. Prz Menopauzalny 13(5):280–288. 10.5114/pm.2014.46470 26327867 10.5114/pm.2014.46470 PMC4520377

[35] Hartini H, Vlorensia, Abdullah H, Martinus AR, Ikhtiari R (2019) The effect of a moisturizing cream containing saccharide isomerates and ceramide on reducing transepidermal water loss in eczema. SCITEPRESS 411–417

[36] Dewi DA, Pangkahila W (2022) Additional of 5% saccharide isomerates in moisturizing formulation increases skin hydration higher than regular moisturizers. Eduvest J Univ Stud 2(8):1537–1549

[37] Qu Y, Ma WY, Sun Q (2017) The comparison of the rejuvenation effects on the skin of Wistar rats between 10600 Nm CO₂ fractional laser and retinoic acid. Eur Rev Med Pharmacol Sci 21:1952–1958 28485780

